# Association between dietary index for gut microbiota and cardiovascular-kidney-metabolic syndrome: a population-based study

**DOI:** 10.3389/fnut.2025.1594481

**Published:** 2025-07-30

**Authors:** Dan Long, Chenhan Mao, Haoyu An, Ying Zhu, Yin Xu

**Affiliations:** ^1^Department of Gastroenterology, The First Hospital of Hunan University of Chinese Medicine, Changsha, China; ^2^Department of Cardiology, Affiliated Hospital of Integrated Traditional Chinese and Western Medicine, Nanjing University of Chinese Medicine, Nanjing, China; ^3^Population Health Sciences Institute, Newcastle University, Newcastle upon Tyne, United Kingdom

**Keywords:** dietary index for gut microbiota, cardiovascular-kidney-metabolic syndrome, gut microbiota diversity, NHANES, cross-sectional study

## Abstract

**Background:**

Cardiovascular-kidney-metabolic (CKM) syndrome represents a major health threat globally. The newly proposed dietary index for gut microbiota (DI-GM), which quantifies dietary quality associated with gut microbiota diversity, may influence the risk of CKM syndrome. Therefore, this study examined the correlation between DI-GM and the prevalence of CKM syndrome, aiming to provide insights for preventive innovation and tailored treatment methods.

**Methods:**

This cross-sectional study included 8,400 adults aged 20 years and older from the National Health and Nutrition Examination Survey (NHANES) 2007–2018. The potential association between the DI-GM score and CKM syndrome was evaluated using univariable and multivariable weighted logistic regression models, restricted cubic spline (RCS), and subgroup analyses.

**Results:**

The average age of the participants was 45.5 years, with 52.0% of the participants being male. A higher DI-GM score was significantly associated with a lower prevalence of CKM syndrome (OR = 0.87, 95% CI: 0.81 to 0.92, *p* <  0.001). The RCS analysis further confirmed a linear relationship between DI-GM score and CKM syndrome (*p* for nonlinear = 0.194). Furthermore, subgroup analysis indicated that sex potentially influenced the association between DI-GM and CKM syndrome (*p* for interaction = 0.004), with the protective effect being more pronounced among U. S. females.

**Conclusion:**

DI-GM score exhibits an inverse correlation with the risk of CKM syndrome. Optimizing dietary patterns to improve DI-GM is associated with reduced risk of CKM syndrome.

## 1 Introduction

The American Heart Association (AHA) defines cardiovascular-kidney-metabolic (CKM) syndrome as a multistage, multisystem disorder (1). CKM syndrome highlights the complex interactions between cardiovascular, renal, and metabolic dysfunctions. Recent epidemiological data reveal a marked increase in the prevalence of CKM syndrome, primarily attributable to demographic shifts including aging populations and the growing burden of lifestyle-related risk factors (2, 3). A cross-sectional study found that more than half of adults in the United States are affected by CKM syndrome (2). CKM syndrome is classified into five stages (0–4), with advancing stages correlating with increased severity of multi-organ dysfunction and poorer prognosis. In addition to increasing the risk of multiorgan failure, CKM syndrome also significantly boosts the likelihood of adverse cardiovascular events and death (4, 5). Consequently, early diagnosis and comprehensive management are essential to modify disease progression and improve clinical outcomes in CKM syndrome.

The involvement of gut microbiota (GM) in metabolic disorders, CVD, and chronic kidney disease (CKD) has attracted much attention in recent years (6–8). It has been suggested that GM dysbiosis may result in chronic inflammation, insulin resistance (IR), and vascular endothelial dysfunction, all of which are core pathologic features of CKM syndrome (9, 10) Trimethylamine N-oxide (TMAO), a GM metabolite derived from dietary levulinic acid and choline, is strongly related to atherosclerosis and increased risk of cardiovascular events (11). Beneficial metabolites, such as short-chain fatty acids (SCFAs), provide a protective effect against metabolic syndrome (MetS) and CKD through their anti-inflammatory and immunomodulatory actions (12, 13).

Numerous studies have demonstrated that dietary patterns exert profound modulatory effects on the composition and diversity of GM (14). High-fat diets worsen metabolic disorders and cardiovascular damage by disrupting the intestinal barrier, decreasing the quantity of helpful bacteria, and increasing the colonization of harmful microbes (15, 16). Conversely, diets rich in dietary fiber regulate the composition and diversity of GM, stimulate the generation of SCFAs, improve IR, and reduce the risk of CKM syndrome (17–19). Dietary intervention represents a translatable strategy to potentially decrease CKM syndrome risk. However, the majority of recent research focuses on the effects of single dietary components or GM metabolites. Methods to systematically evaluate the overall impact of dietary patterns on GM are lacking. The newly proposed dietary index of gut microbiota (DI-GM) quantifies dietary quality associated with GM diversity (20). Higher DI-GM scores represent healthier GM.

Although studies have demonstrated associations between DI-GM and several diseases (21), its relevance to CKM syndrome remains inadequately explored. Consequently, this study aims to assess the association between DI-GM scores and the prevalence of CKM syndrome using cross-sectional data from the National Health and Nutrition Examination Survey (NHANES) conducted between 2007 and 2018. These findings provide a critical foundation for developing targeted prevention strategies and personalized dietary interventions in CKM syndrome management.

## 2 Methods

### 2.1 Study design and participants

**Figure 1:**
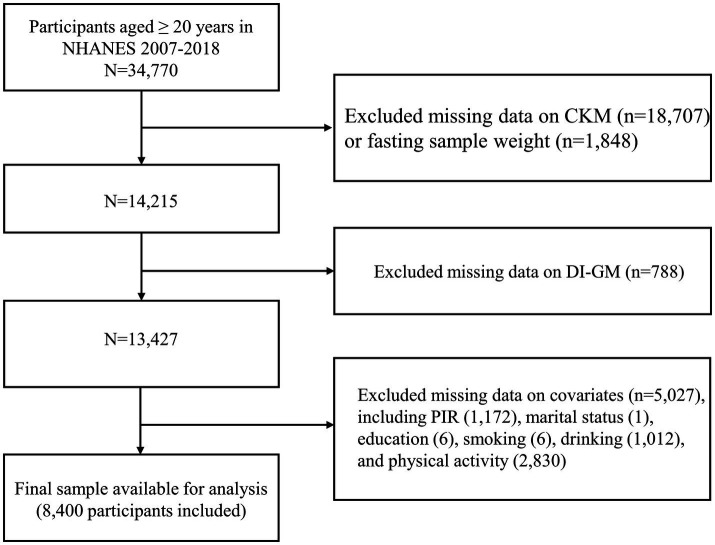
The flowchart for population screening. CKM, cardiovascular-kidney-metabolic syndrome; DI-GM, dietary index for gut microbiota; PIR, family income-to-poverty ratio.

NHANES utilized a sophisticated, stratified, multistage probability sampling design to ensure a nationally representative sample (22). The raw data analyzed in this study were obtained directly from the publicly available NHANES database.^1^ This study involved a total of 34,770 adults aged 20 years and above, drawing data from six NHANES cycles between 2007 and 2018. After a rigorous screening procedure, participants lacking diagnostic data on CKM syndrome (*n* = 18,707), those missing sample weights (*n* = 1,848), and individuals without data on DI-GM components (*n* = 788) were excluded. In addition, we excluded participants with incomplete information on covariates (*n* = 5,027). The final analysis included 8,400 eligible participants (Figure 1).

### 2.2 Calculation of DI-GM

DI-GM was calculated based on the intake of 14 foods or nutrients using the scoring system developed by Kase et al. (20). Avocados, broccoli, chickpeas, coffee, cranberries, fermented dairy products, dietary fiber, green tea (unavailable due to NHANES not recording the specific type of tea consumption), soy, and whole grains were categorized as beneficial ingredients. In contrast, red meat, processed meats, refined grains, and high-fat diets (≥ 40% of total energy intake) were identified as harmful elements. The DI-GM score was calculated using 24-h dietary recall data from NHANES. For beneficial ingredients, a score of 1 was assigned if the intake was at or above the sex-specific median; otherwise, a score of 0 was given. For harmful components, a score of 0 was assigned if the intake was at or above the sex-specific median (or if ≥ 40% of energy was derived from fat); otherwise, a score of 1 was given. The DI-GM score is the sum of the scores for all components, ranging from 0 to 13.

Given the objective of this study, the DI-GM score was defined as the exposure variable. The DI-GM score was categorized into four groups (0–3, 4, 5, and ≥ 6) based on both established literature and our data distribution characteristics. This categorization aligns with previously published NHANES studies investigating DI-GM (21, 23). The thresholds were biologically meaningful and clinically relevant: scores ≤ 3 represent inadequate dietary support for GM, 4–5 indicate moderate support, and ≥ 6 reflect optimal dietary patterns for microbial health. Importantly, these cutpoints closely correspond to key percentiles in our data distribution - the 1st quartile (4), median (5), and 3rd quartile (6) - allowing for balanced group sizes while maintaining clinical interpretability.

### 2.3 Assessment of CKM stages

Participants were categorized into those without CKM syndrome (CKM syndrome stage 0) and patients suffering from CKM syndrome (CKM stages 1–4). The stages of CKM syndrome were defined according to the 2023 AHA Presidential Recommendation on CKM Health (1). Individuals in CKM stage 0 have no CKM-related risk factors. Stage 1 encompasses participants exhibiting either obesity or impaired glucose metabolism. Obesity is defined as a body mass index (BMI) ≥ 23 kg/m^2^ for individuals of Asian race and >25 kg/m^2^ for all other races and ethnic groups. Prediabetes is defined as a glycated hemoglobin (HbA1c) of 5.7% to < 6.5% or a fasting blood glucose of 100 mg/dL to < 126 mg/dL. Participants in CKM stage 2 have developed metabolic risk factors (such as elevated fasting serum triglycerides (≥ 135 mg/dL), hypertension, diabetes, and MetS) or moderate to severe CKD. Hypertension was defined as systolic blood pressure ≥ 130 mmHg, diastolic blood pressure ≥ 80 mmHg, or antihypertensive medication use. Diabetes was defined as HbA1c ≥ 6.5%, self-reported physician diagnosis, or antidiabetic medication use. MetS is diagnosed when an individual meets ≥ 3 of the following criteria: increased waist circumference, low high-density lipoprotein cholesterol level (< 40 mg/dL or < 50 mg/dL for men or women, respectively), fasting triglycerides ≥ 150 mg/dL, elevated blood pressure, or prediabetes. CKD stages were defined according to the estimated glomerular filtration rate [eGFR, calculated with the race-free CKD-EPI 2021 creatinine equation (24)] and urinary albumin to creatinine ratio (UACR). Moderate to severe CKD was identified by eGFR < 60 mL/min/1.73 m^2^ or UACR > 30 mg/g. Patients in CKM stage 3 have combined subclinical CVD. Subclinical CVD was defined as a 10-year CVD risk of 20% or more as predicted by the basic PREVENT equation (25). Participants in CKM stage 4 had been diagnosed with CVD, including coronary heart disease, angina, myocardial infarction, heart failure, and cerebrovascular accident. CKM syndrome was considered an outcome variable in this study. The history of CVD was assessed through validated self-reports of physician diagnoses.

### 2.4 Covariates

In line with previous studies, this study incorporated potential covariates to account for possible confounding factors (26). The covariates comprised sociodemographic, socioeconomic, and health behavior factors. BMI was not included as a covariate because body weight status is inherently embedded in the CKM staging system. Adjusting for BMI would statistically over-correct for a component already reflected in the outcome definition. Sociodemographic and socioeconomic factors included age, sex, race/ethnicity, marital status, educational level, and the poverty income ratio (PIR). Health-related behaviors encompassed smoking status, alcohol consumption, energy intake, and physical activity. Detailed assessment criteria for smoking, drinking status, and physical activity are presented in Supplementary Table 1.

### 2.5 Statistical analysis

This study utilized sample weights in all analyses to guarantee nationally representative results. For continuous variables, participant characteristics were presented as weighted means with standard errors (SE). For categorical variables, counts with weighted percentages were reported. Depending on the distribution of the data, differences in baseline characteristics between groups were compared using Student’s t-test, Wilcoxon rank-sum test, Kruskal-Wallis test, or chi-square test. Associations of DI-GM score and other covariates with the risk of CKM syndrome were evaluated by univariable weighted logistic regression. Three multivariable weighted logistic regression models were utilized to calculate the adjusted odds ratios (ORs) for DI-GM scores, as well as their corresponding 95% confidence intervals (CIs). In Model 1, no covariates were adjusted. Model 2 was adjusted for age, sex, and race/ethnicity. Model 3 was further adjusted for potential confounders, including age, sex, race/ethnicity, education, marital status, PIR, smoking, drinking, energy intake, and physical activity. Additionally, a restricted cubic spline (RCS) model with four knots was applied to determine the dose–response correlation between DI-GM scores and the risk of CKM syndrome. The RCS model was adjusted for age, sex, race/ethnicity, education, marital status, PIR, smoking, drinking, energy intake, and physical activity. Subgroup analyses and interaction tests were performed to explore possible subgroup-specific associations. Sensitivity analyses were conducted to validate the robustness of the results. Missing data for covariates were imputed, and the aforementioned analyses were repeated using the imputed dataset (27).

All statistical analyses were performed using R software (version 4.3.2). The “survey” package was used for weighted analyses. In sensitivity analyses, missing values for covariates were handled using multiple imputation with the “mice” package in R, employing a random forest algorithm. A *p*-value < 0.05 was considered statistically significant.

## 3 Results

### 3.1 Baseline population characteristics

The final analysis comprised 8,400 eligible adults aged 20 and older, 7,590 of whom had CKM syndrome. Consistent with previous studies (2), we found that nearly 90% of US adults met criteria for CKM syndrome, which may be related to the broad definition of CKM syndrome. As shown in Table 1, the proportions of Stage 1, Stage 2, Stage 3, and Stage 4 populations within the total study population were 24.5, 53.9, 2.4, and 7.9%, respectively. The average age of participants was 45.5 years, with 52.0% being male. Compared to participants without CKM syndrome, those diagnosed with CKM syndrome were significantly older (*p* < 0.001). Participants had a mean DI-GM score of 4.73 ± 0.03. Notably, participants diagnosed with CKM syndrome (4.69 ± 0.03) had significantly lower DI-GM scores than those without CKM syndrome (5.02 ± 0.07) (*p* < 0.001). Compared to non-CKM syndrome participants, individuals with CKM syndrome tend to be older (*p* < 0.001), more likely to be male (*p* < 0.001), have lower income levels (*p* = 0.008), and attain less education (*p* < 0.001). Moreover, they are also more likely to have a history of smoking or alcohol consumption and to be less physically active (*p* < 0.001). Supplementary Table 2 shows the baseline characteristics of participants categorized by their DI-GM scores. Participants with higher PIR and higher educational attainment were more likely to have higher DI-GM scores (*p* < 0.001). Compared to participants with lower DI-GM scores, those with higher DI-GM scores were more likely to have lower BMI (*p* < 0.001), HbA1c (*p* = 0.011), and serum creatinine levels (*p* = 0.024).

**Table 1:** Survey-weighted baseline characteristics of participants stratified by CKM syndrome.

Characteristics	Total (*n* = 8,400)	Non-CKM syndrome (*n* = 810)	CKM syndrome (*n* = 7,590)	*p*-value
Age, mean (SE), years	45.5 (0.3)	34.2 (0.5)	47.0 (0.3)	<0.001
Sex, *n* (weighted %)				<0.001
Female	4,021 (48.0)	520 (64.9)	3,501 (45.8)	
Male	4,379 (52.0)	290 (35.1)	4,089 (54.2)	
Race, *n* (weighted %)				<0.001
Mexican American	1,210 (7.7)	82 (5.0)	1,128 (8.1)	
Non-Hispanic Black	1,602 (10.2)	96 (6.7)	1,506 (10.7)	
Non-Hispanic White	3,810 (70.0)	419 (75.1)	3,391 (69.3)	
Other Race	1,778 (12.1)	213 (13.3)	1,565 (11.9)	
PIR, mean (SE)	3.10 (0.04)	3.29 (0.07)	3.08 (0.05)	0.008
BMI, mean (SE), kg/m^2^	28.69 (0.12)	21.68 (0.10)	29.62 (0.11)	<0.001
Educational level, *n* (weighted %)				<0.001
Less than high school	579 (3.4)	24 (1.4)	555 (3.6)	
High school or equivalent	2,897 (31.2)	202 (22.2)	2,695 (32.3)	
College or above	4,924 (65.5)	584 (76.4)	4,340 (64.0)	
Marital status, *n* (weighted %)				<0.001
Widowed/Divorced/Never married	3,289 (35.5)	398 (43.0)	2,891 (34.5)	
Married/Living with a partner	5,111 (64.5)	412 (57.0)	4,699 (65.5)	
Smoking status, *n* (weighted %)				<0.001
Never	4,672 (56.0)	575 (69.8)	4,097 (54.1)	
Former	2,058 (25.3)	93 (12.8)	1,965 (26.9)	
Now	1,670 (18.8)	142 (17.4)	1,528 (18.9)	
Alcohol drinking, *n* (weighted %)				<0.001
Never	972 (9.0)	94 (8.9)	878 (9.0)	
Former	1,128 (10.8)	52 (5.5)	1,076 (11.5)	
Mild	3,083 (39.3)	305 (39.8)	2,778 (39.2)	
Moderate	1,378 (18.3)	173 (22.8)	1,205 (17.7)	
Heavy	1,839 (22.6)	186 (23.0)	1,653 (22.5)	
Physical activity, *n* (weighted %)				<0.001
Mild	1,548 (16.9)	111 (11.4)	1,437 (17.6)	
Moderate	3,376 (41.2)	356 (45.7)	3,020 (40.6)	
Vigorous	3,476 (41.9)	343 (42.9)	3,133 (41.8)	
Hypertension, *n* (weighted %)				<0.001
No	5,145 (65.5)	810 (100.0)	4,335 (61.0)	
Yes	3,255 (34.5)	0 (0.0)	3,255 (39.0)	
Diabetes, *n* (weighted %)				<0.001
No	6,898 (86.9)	810 (100.0)	6,088 (85.1)	
Yes	1,502 (13.1)	0 (0.0)	1,502 (14.9)	
CVD, *n* (weighted %)				<0.001
No	7,650 (92.7)	810 (100.0)	6,840 (91.8)	
Yes	750 (7.3)	0 (0.0)	750 (8.2)	
CKD, *n* (weighted %)				<0.001
No	7,204 (88.7)	810 (100.0)	6,397 (87.2)	
Yes	1,196 (11.3)	0 (0.0)	1,193 (12.8)	
Total energy intake, mean (SE), kcal	2,237.62 (13.02)	2,211.61 (38.80)	2,241.06 (14.59)	0.499
SBP, mean (SE), mmHg	120.16 (0.26)	107.56 (0.36)	121.82 (0.26)	<0.001
DBP, mean (SE), mmHg	70.10 (0.24)	64.11 (0.41)	70.89 (0.24)	<0.001
WBC, mean (SE), 103/uL	6.70 (0.04)	6.00 (0.07)	6.79 (0.04)	<0.001
HGB, mean (SE), g/dL	14.42 (0.03)	14.11 (0.06)	14.46 (0.03)	<0.001
FBG, mean (SE), mg/dL	104.79 (0.42)	90.31 (0.25)	106.70 (0.45)	<0.001
HbA1c (%)	5.56 (0.01)	5.15 (0.01)	5.62 (0.01)	<0.001
TC, mean (SE), mmol/L	4.98 (0.02)	4.56 (0.04)	5.03 (0.02)	<0.001
TG, mean (SE), mg/dL	120.95 (1.49)	68.47 (1.18)	127.90(1.60)	<0.001
LDL, mean (SE), mmol/L	2.96 (0.01)	2.56 (0.04)	3.02 (0.01)	<0.001
SCR, mean (SE), umol/L	77.09 (0.34)	71.81 (0.57)	77.78 (0.37)	<0.001
eGFR, mean (SE), ml/min/1.73 m^2^	98.01 (0.34)	106.01 (0.73)	96.95 (0.34)	<0.001
UACR, mean (SE), mg/g	25.50 (2.05)	7.68 (0.28)	27.86 (2.31)	<0.001
ALT, mean (SE), U/L	25.32 (0.23)	19.72 (0.44)	26.07 (0.25)	<0.001
AST, mean (SE), U/L	24.97 (0.20)	23.14 (0.41)	25.22 (0.22)	<0.001
GGT, mean (SE), U/L	26.86 (0.46)	16.09 (0.39)	28.29 (0.51)	<0.001
DI-GM score, mean (SE)	4.73 (0.03)	5.02 (0.07)	4.69 (0.03)	<0.001
DI-GM group, *n* (weighted %)				0.001
0–3	1,968 (22.4)	161 (17.6)	1,807 (23.0)	
4	2,062 (23.4)	194 (21.6)	1,868 (23.6)	
5	1,935 (23.1)	186 (23.7)	1,749 (23.1)	
≥ 6	2,435 (31.1)	269 (37.2)	2,166 (30.3)	

Continuous variables are described as weighted means (standard errors). For categorical variables, unweighted N reflects the study sample, while percentages represent survey-weighted values. CKM, cardiovascular-kidney-metabolic; SE, standard error; PIR, poverty income ratio; BMI, body mass index; CVD, cardiovascular disease; CKD, chronic kidney disease; SBP, systolic blood pressure, DBP, diastolic blood pressure; WBC, white blood cell; HGB, hemoglobin; FBG, fasting blood glucose; HbA1c, glycated hemoglobin; TC, total cholesterol; TG, fasting triglyceride; LDL, low-density lipoprotein; SCR, serum creatinine; eGFR, estimated glomerular filtration rate; UACR, urinary albumin creatinine ratio; ALT, alanine transaminase; AST, aspartate transaminase; GGT, gamma-glutamyltransferase; DI-GM, dietary index for gut microbiota.

### 3.2 Risk factors for CKM syndrome

To further investigate the underlying risk factors for CKM syndrome, univariable weighted logistic regression models were performed to analyze multiple potentially influential factors. The results are reported in Supplementary Table 3. Potential risk factors for CKM syndrome included advanced age, male sex, poverty, smoking, alcohol intake, and inadequate exercise (*p* < 0.05). Notably, DI-GM may be a key protective factor for CKM syndrome (*p* < 0.001). Therefore, to exclude confounding factors and further examine the relationship between DI-GM score and CKM syndrome risk, we adjusted for potential covariates by multivariable weighted logistic regression analysis and evaluated nonlinear associations by RCS analysis. The results indicated that a lower DI-GM score served as an independent risk factor for the onset of CKM syndrome (*p* < 0.001).

### 3.3 Association between DI-GM and CKM syndrome risk

**Figure 2:**
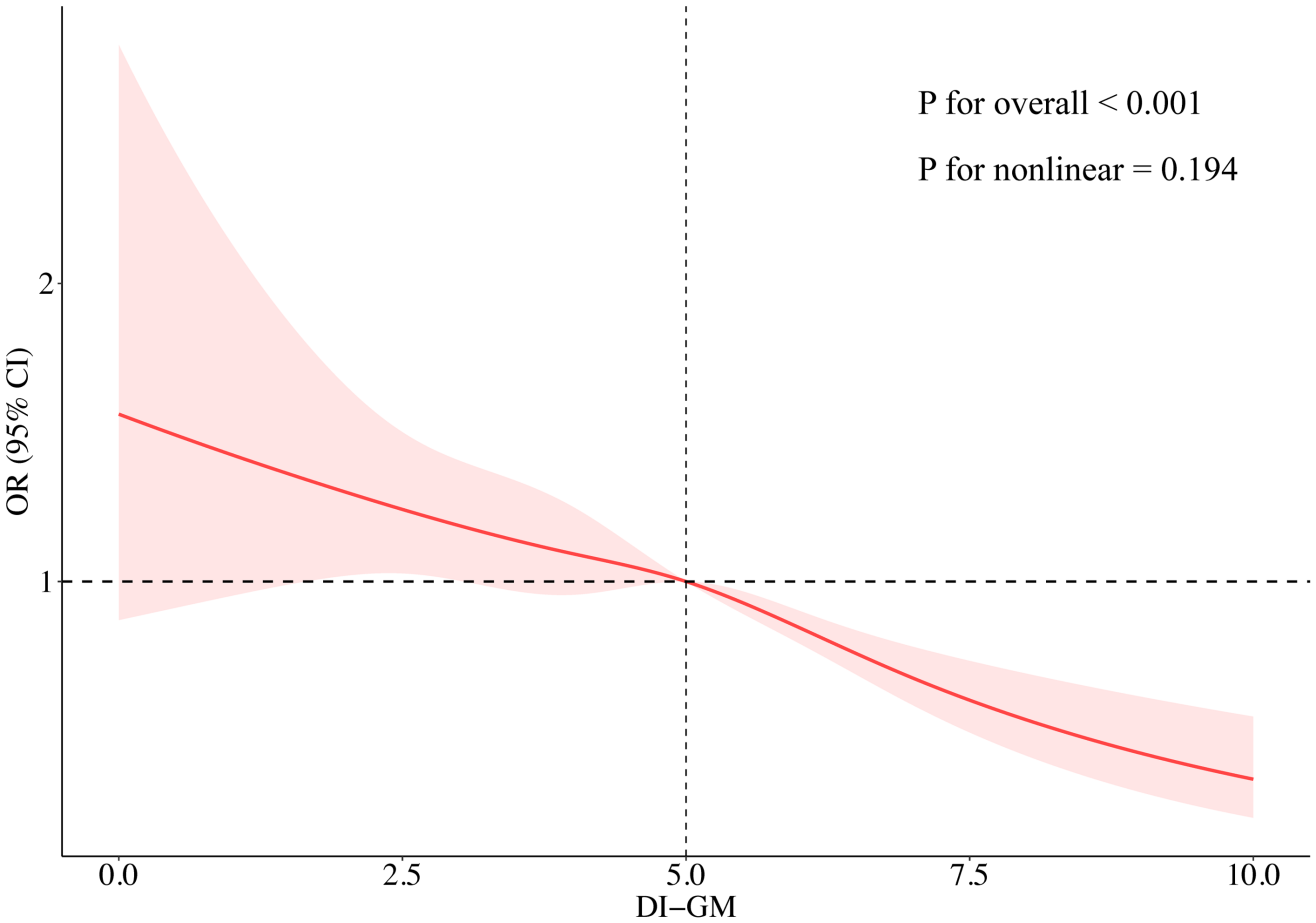
Association between DI-GM and the risks of CKM syndrome analyzed through a restricted cubic spline model. The restricted cubic spline analysis was adjusted for age, sex, race/ethnicity, marital status, education, PIR, alcohol intake, smoking, physical activity, and total energy intake. DI-GM, dietary index for gut microbiota; CKM, cardiovascular-kidney-metabolic; OR, odds ratio; CI, confidence interval.

The results of multivariable logistic regression analysis demonstrated a significant association between DI-GM scores and the prevalence of CKM syndrome. As presented in Table 2, a significant inverse correlation was detected between DI-GM scores and CKM syndrome in model 1 without covariate adjustment (OR = 0.89, 95% CI: 0.84 to 0.93, *p* < 0.001). The inverse correlation persisted consistently after accounting for multiple potential confounders in model 3 (OR = 0.87; 95% CI: 0.81 to 0.92, *p* < 0.001). Compared to participants with DI-GM scores of 0–3, those with DI-GM scores of 6 or higher had a 43% lower prevalence of CKM syndrome (Model 3: OR = 0.57, 95% CI: 0.44 to 0.75, *p* < 0.001). A significant decreasing trend in CKM syndrome risk was observed with increasing DI-GM scores (*p* for trend < 0.001). After adjusting for all covariates in model 3, the RCS result revealed a linear association between DI-GM score and CKM syndrome (*p* for nonlinear = 0.194, Figure 2).

**Table 2:** The association between DI-GM and CKM syndrome among adults in NHANES 2007–2018.

Characteristics	Model 1		Model 2		Model 3	
	OR (95% CI)	*p*-value	OR (95% CI)	*p*-value	OR (95% CI)	*p*-value
DI-GM score	0.89 (0.84, 0.93)	<0.001	0.84 (0.79, 0.89)	<0.001	0.87 (0.81,0.92)	<0.001
DI-GM group						
0–3	Reference	Reference	Reference	Reference	Reference	Reference
4	0.84 (0.64, 1.09)	0.176	0.79 (0.59, 1.06)	0.116	0.82 (0.61, 1.10)	0.184
5	0.74 (0.58, 0.95)	0.017	0.70 (0.54, 0.91)	0.008	0.76 (0.58, 0.99)	0.041
≥ 6	0.62 (0.49, 0.78)	<0.001	0.51 (0.39, 0.66)	<0.001	0.57 (0.44, 0.75)	<0.001
Trend test		<0.001		<0.001		<0.001

Model 1 was not adjusted for any covariates. Model 2 was adjusted for age, sex, and race/ethnicity. Model 3 was adjusted for age, sex, race/ethnicity, marital status, education level, PIR, alcohol intake, smoking status, physical activity, and total energy intake. DI-GM, dietary index for gut microbiota; CKM, cardiovascular-kidney-metabolic; NHANES, National Health and Nutrition Examination Survey; OR, odds ratio; CI, confidence interval; PIR, poverty income ratio.

### 3.4 Subgroup analysis and sensitivity analysis

**Figure 3:**
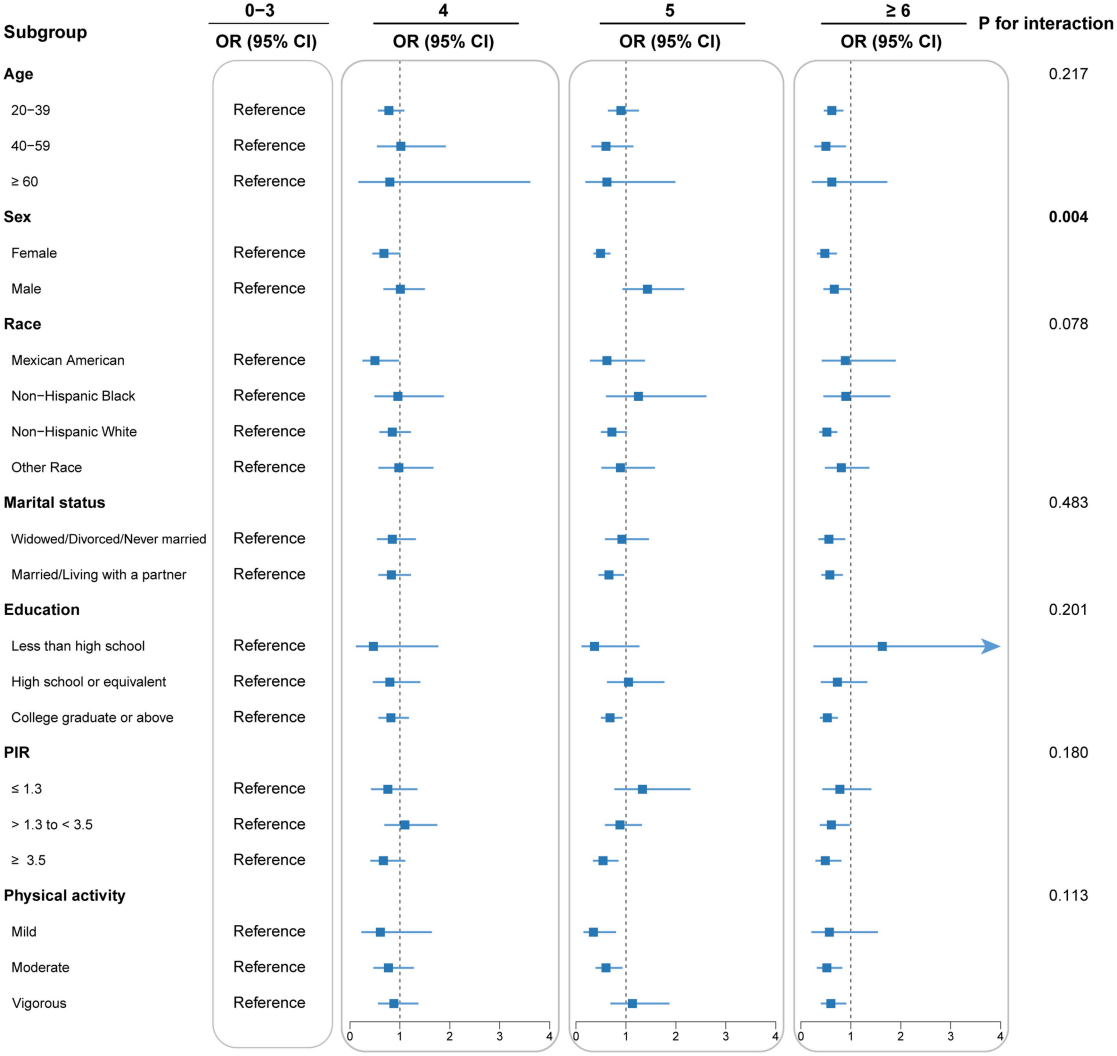
Subgroup analysis for the association between DI-GM and CKM syndrome. Each stratification was adjusted for age, sex, race, marital status, educational levels, poverty income ratio, smoking behavior, alcohol consumption, physical activity, and total energy intake, unless the variable was already used as a stratification factor. DI-GM, dietary index for gut microbiota; CKM, cardiovascular-kidney-metabolic; OR, odds ratio; CI, confidence interval; PIR, poverty income ratio.

Subgroup analyses and interaction tests were conducted to detect potential heterogeneity among diverse population subgroups. Subgroup analyses accounted for all covariates excluding stratification variables. As shown in Figure 3, the inverse correlation between DI-GM score and CKM syndrome remains consistent across various subgroups, including age, race, education, PIR, marital status, and physical activity (*p* for interaction > 0.05). Due to multiple comparisons in the subgroup analyses, *p*-values were adjusted for multiplicity (Supplementary Table 4). A significant interaction was identified between DI-GM score and sex subgroups (*p* for interaction = 0.004), indicating that the influence of DI-GM on CKM syndrome potentially differs by sex. Notably, the correlation was significantly stronger in females than males. False-positive results may arise from multiple subgroup analyses despite multiple testing adjustments. These findings need to be validated in independent samples. Moreover, missing values of all covariates were multiply interpolated for sensitivity analysis. In the sensitivity analysis, multivariable logistic regression showed a stable and significant inverse association between DI-GM score and CKM syndrome (Supplementary Table 5). The RCS model showed a linear connection between DI-GM score and CKM syndrome (*p* for nonlinear = 0.246; Supplementary Figure 1).

### 3.5 Development and validation of the prediction nomogram

**Figure 4:**
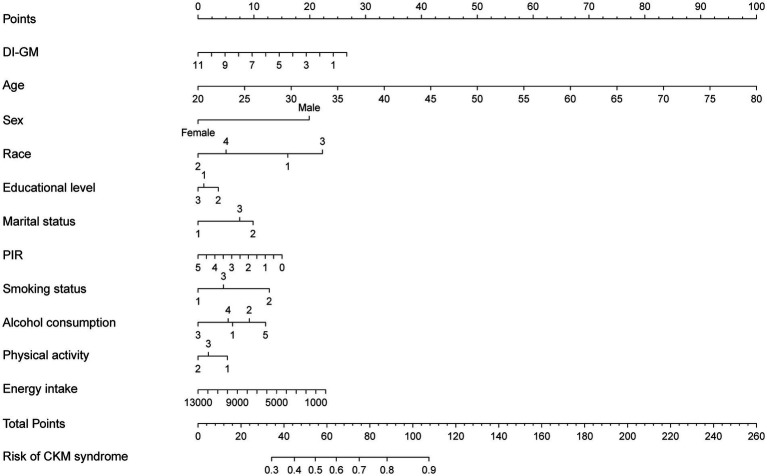
A nomogram model for predicting the risk of CKM syndrome. The developed nomogram model was based on model 3, including DI-GM, age, sex, race, marital status, education level, PIR, alcohol intake, smoking status, physical activity, and total energy intake. The categorical variables are coded as follows. Race: 1 = Mexican American; 2 = Non-Hispanic White; 3 = Non-Hispanic Black; 4 = other race. Educational level: 1 = less than high school; 2 = high school or equivalent; 3 = college graduate or above. Marital status: 1 = never married; 2 = married/living with partner; 3 = divorced/widowed/separated. Smoking status: 1 = never; 2 = former; 3 = current. Alcohol consumption: 1 = never; 2 = former; 3 = mild; 4 = moderate; 5 = heavy. Physical activity: 1 = mild; 2 = moderate; 3 = vigorous. CKM, cardiovascular-kidney-metabolic; DI-GM, dietary index for gut microbiota; PIR, poverty income ratio.

**Figure 5:**
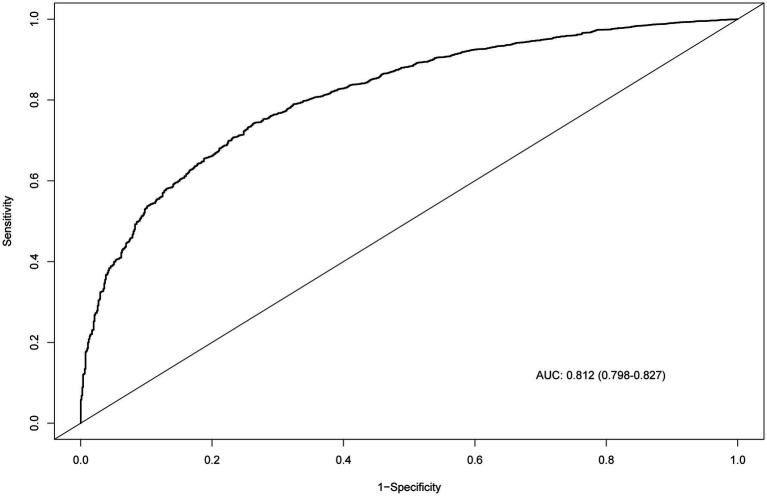
ROC curve evaluating the predictive power of the nomogram model. ROC, receiver operating characteristic; AUC, area under the curve.

The nomogram for predicting CKM syndrome risk was constructed using the variables in Model 3. Points are assigned to each factor based on its position along the respective scale (Figure 4). The total score is calculated by summing all individual points. Then, draw a vertical line from the total points to the “Risk of CKM syndrome” scale to estimate the predicted risk. We assessed the nomogram’s predictive accuracy using receiver operating characteristic (ROC) curve analysis (Figure 5). The nomogram showed good predictive power, with an area under the ROC curve (AUC) of 0.812 (95% CI, 0.798–0.827).

## 4 Discussion

This study conducted a national cross-sectional examination of the relationship between DI-GM and CKM syndrome using NHANES data from 2007 to 2018. DI-GM serves as a scientifically validated tool to evaluate how dietary patterns affect health through GM. Unlike previous studies that focused on isolated conditions (28, 29), the present study links DI-GM to multi-organ dysfunction. We found that the DI-GM score exhibited an inverse linear relationship with CKM syndrome risk. This inverse correlation remains significant even after comprehensive adjustment for covariates in model 3 (OR = 0.87, 95% CI: 0.81 to 0.92), which parallels DI-GM’s relationships on CKD (OR = 0.96, 95% CI: 0.94 to 0.98) (29) and metabolic dysfunction-associated fatty liver (OR = 0.94, 95% CI: 0.90 to 0.98) (23). Subgroup analyses suggested that the inverse association between DI-GM and CKM syndrome was stronger in women. Optimizing dietary patterns to improve DI-GM may be associated with a lower risk of CKM syndrome.

The identified inverse correlation between DI-GM and CKM syndrome risk is consistent with previous studies that reveal the significant role of GM diversity in CKM syndrome pathogenesis (9, 30). Numerous studies have identified GM dysbiosis in patients with CKM syndrome, characterized by an increase in pathogenic bacterial species and a reduction in the abundance of beneficial microorganisms (31–33). For example, GM diversity decreased with increasing 10-year CVD risk and BMI (34). GM dysbiosis drives the development and progression of CKD. Substantial evidence indicates that elevated levels of GM-derived harmful metabolites, including TMAO, indoxyl sulfate, and p-cresyl sulfate, are strongly related to various adverse clinical outcomes in CKD patients (35). The accumulation of these metabolites correlates with accelerated renal fibrosis, endothelial dysfunction, and a decrease in estimated glomerular filtration rate, resulting in a notable rise in the incidence and mortality of cardiovascular complications.

The diet significantly affects the diversity and function of GM (36). The Western dietary pattern features refined grains, red meat, processed meat, high-sugar foods, and high-fat dairy products. A meta-analysis indicated that the intake of both unprocessed and processed red meat contributes to an elevated risk of CVD, its subtypes, and diabetes, with more pronounced associations observed in Western populations (37). A low DI-GM diet may lead to GM dysbiosis, triggering inflammatory responses and increasing the risk of CKM syndrome (38, 39). Additionally, high-fat diets, particularly those rich in saturated fats, can cause microbial dysbiosis and increased intestinal permeability. High-fat diets reduce the levels of SCFAs, including acetate, 2-hydroxybutyrate, and 2-methylbutyrate (40, 41). Conversely, the Mediterranean diet, abundant in whole grains, vegetables, fruits, legumes, nuts, and olive oil, results in a significant reduction in the risk of CKM syndrome (42, 43). Evidence demonstrated that compliance with the Mediterranean diet is negatively correlated with the occurrence and death of CVD and significantly lowers blood pressure (44, 45). The benefits of the Mediterranean diet are attributed to its higher consumption of fiber, polyphenols, and unsaturated fatty acids, which regulate the GM and enhance metabolic health.

The association between DI-GM and the development of CKM syndrome could involve various mechanisms. GM may influence the onset and course of CKM syndrome by regulating metabolite production, inflammatory responses, and immune homeostasis. The abnormal accumulation and malfunction of adipose tissue, especially visceral adipose tissue, are primary contributors to the excessive release of pro-inflammatory factors (46). Individuals with CKM syndrome frequently endure a sustained low-grade inflammatory condition. Persistent inflammation triggers IR and endothelial dysfunction, exacerbating the development of atherosclerosis and ultimately affecting critical organs such as the heart and kidneys (47). Almonds are rich in fiber, monounsaturated fatty acids, and *α*-tocopherol. They have been found to inhibit cholesterol synthesis and improve endothelial dysfunction (48). Furthermore, a high DI-GM dietary pattern can enhance the proliferation of advantageous bacteria, including *Bifidobacterium* and *Lactobacillus* (49, 50). These beneficial bacteria can modulate the intestinal immune system and suppress the production of pro-inflammatory cytokines. For example, *Bifidobacterium* can regulate the Toll-like receptor signaling pathway and inhibit the activation of nuclear factor kappa B, thereby reducing the expression of pro-inflammatory cytokines such as tumor necrosis factor-alpha and interleukin-6 (51).

GM is involved in the metabolic processes of a wide range of substances, and its metabolites have profound effects on host health. SCFAs, which are generated by the degradation of dietary fiber and carbohydrates, play a significant role in the immune and metabolic systems (52). High DI-GM diets enhance the secretion of SCFAs, including acetate, propionate, and butyrate (53). An almond-based diet may effectively enhance the abundance of SCFAs-producing bacteria while reducing glycated hemoglobin and BMI among those with type 2 diabetes (54). Supplementation with SCFA-producing bacteria (such as *Lactobacillus*) or following a Mediterranean diet can considerably improve blood pressure and dyslipidemia (55). Interestingly, SCFAs regulate blood pressure via G protein-coupled receptor 41 and olfactory receptor 78 (56, 57). Butyrate reduces blood lipid levels and improves atherosclerosis (58). Furthermore, intestinal endocrine cells may release glucagon-like peptide-1 (GLP-1) in response to SCFAs (59). GLP-1 stimulates insulin secretion and improves IR, effectively improving the abnormal glucose metabolism common in CKM syndrome.

Interestingly, subgroup analysis indicated that the inverse correlation between DI-GM scores and CKM syndrome was more significant in women. These findings underscore the importance of recognizing sex differences in the management of CKM syndrome risk factors. There could be several mechanisms underlying the observed sex differences. First, estrogen levels in women may enhance the metabolic protective effects of dietary interventions by increasing the abundance of SCFA-producing bacteria (60). Reduced estrogen levels are closely associated with GM dysbiosis and chronic low-grade inflammation (60). Previous research has demonstrated that estrogen receptor *β* positively regulates GM diversity (61). Additionally, estrogen-related receptors can improve mitochondrial function by activating the PPARG coactivator (PGC)-1α pathway (62). The microbial metabolite indole-3-propionic acid has been shown to specifically interact with the PGC-1α pathway, upregulating genes related to mitochondrial biogenesis and antioxidant responses, thereby delaying the progression of diabetic kidney disease (62). Second, sex-specific differences in GM composition may contribute to this association (63). Studies have found that the abundance of *Lactobacillus*, *Bacteroides*, and *Bifidobacterium* is generally higher in females than males, which may enable females to produce protective metabolites such as butyrate more efficiently through dietary fiber metabolism (64, 65). Last but not least, sex differences in dietary behaviors and adherence may also play a role. Women may exhibit better adherence to and persistence with healthy diets, thereby amplifying the protective effects against CKM syndrome.

In conclusion, interventions targeting GM potentially open new directions in the management of CKM syndrome. These findings stress the importance of dietary strategies designed for enhancing GM health. Dietary patterns abundant in fiber (including whole grains, legumes, vegetables, and fruits) and fermented foods mitigate the risk of CKM syndrome. A personalized diet for CKM syndrome combined with probiotic or prebiotic supplementation can enhance the structure and function of GM and improve metabolic markers (66). There is an urgent need to promote specific dietary patterns, such as the Mediterranean diet and the Dietary Approaches to Stop Hypertension (DASH) diet. Plant-based diets and the Mediterranean diet have been demonstrated to increase the generation of favorable bacterial metabolites such as SCFAs while reducing TMAO levels, thereby lowering the risk of CKM syndrome (67–69). Reducing consumption of red meat, refined sugar, and saturated fat inhibits the proliferation of TMAO-producing bacteria and reduces the risk of atherosclerosis and kidney damage.

This study is the first to evaluate DI-GM in the context of CKM syndrome using national U. S. data. Through sex-stratified analyses of NHANES data, we identified significant effect modifications by sex in the DI-GM and CKM syndrome association. These findings underscore the necessity of sex-specific approaches in both clinical management and public health interventions for CKM syndrome. However, several limitations should be acknowledged. First, the cross-sectional design limits causal inference between DI-GM and CKM syndrome. Second, dietary intake data were collected through dietary recall interviews, which may be affected by recall bias. The use of 24-h dietary recall data may not reflect long-term dietary patterns. Moreover, although the DI-GM was developed based on well-established associations between dietary components and GM features, its direct validation against sequencing-derived microbial composition or functional profiles remains lacking. Additionally, limited to the U. S. adult population, the generalizability of these findings to other populations may be limited due to differences in living environments, dietary patterns, and microbiota profiles. Finally, factors like stress, medications, supplement use, and genetic predisposition were not included. Despite accounting for multiple covariates, residual confounding from unmeasured factors may still affect the detected associations. Despite these limitations, this study offers important insights into the potential role of DI-GM in CKM syndrome. Future studies should tackle these limitations by including longitudinal designs, refining dietary assessment methods, and exploring underlying mechanisms to better understand the association between DI-GM and CKM syndrome.

## 5 Conclusion

This study demonstrated an inverse correlation between DI-GM and the risk of CKM syndrome. Dietary interventions to improve GM diversity may be associated with a reduced risk of CKM syndrome. More in-depth research, including prospective studies and randomized controlled trials, is required to investigate the association between dietary patterns, GM, and CKM syndrome. Additional research is needed to confirm the reported sex differences.

## Data Availability

Publicly available datasets were analyzed in this study. This data can be found at: NHANES website (https://www.cdc.gov/nchs/nhanes/).

## Footnotes

^1^ https://www.cdc.gov/nchs/nhanes/
