# Supplementary material for: Association between dietary index for gut microbiota and cardiovascular-kidney-metabolic syndrome: a population-based study
Source: Front Nutr. 2025 Jul 30;12:1594481. doi: 10.3389/fnut.2025.1594481 (PMC12343273; doi:10.3389/fnut.2025.1594481)
Supplement: Supplementary file 1 [file Table_1.docx]

**Supplementary Materials**

**Supplementary Table 1** Definitions of smoking, drinking status, and physical activity.

| Personal behavioral variables | Definition |
| --- | --- |
| Smoking status | Never smokers: Individuals who have smoked fewer than 100 cigarettes throughout their lifetime.​  Former smokers: Those who have smoked more than 100 cigarettes in their lifetime but do not smoke at all now.​  Current smokers: Individuals who have smoked more than 100 cigarettes in their lifetime and either smoke on some days or smoke daily. |
| Alcohol consumption | Never drinkers: consumed < 12 drinks throughout their entire lifetime;  Former drinkers: consumed ≥ 12 drinks within a single year and did not drink last year, or did not drink last year but drank ≥ 12 drinks in lifetime;  Mild drinkers: male: ≤ 2 drinks per day; female: ≤ 1 drinks per day;  Moderate drinkers: male: ≥ 3 drinks per day; female: ≥ 2 drinks per day; or binge ≥ 2 & binge <5;  Heavy drinkers: male: ≥ 4 drinks per day; female: ≥ 3 drinks per day; or binge drinking ≥ 4 drinks on same occasion for females, ≥ 5 drinks on same occasion for males. |
| Physical activity | Physical activity encompassed the time (in minutes) participants spent on various activities per week, including walking, cycling, household chores, work-related tasks, and recreational activities. The metabolic equivalent of task (MET) scores for specific activities were used to assess exercise intensity. Based on the standard scoring criteria of the International Physical Activity Questionnaire (IPAQ), participants were categorized into three levels: mild (<600 MET-minutes/week), moderate (600-3000 MET-minutes/week), and vigorous (≥3000 MET-minutes/week). |

**Supplementary Table 2** Survey‐weighted baseline characteristics of participants stratified by DI-GM score.

| Variables | DI-GM | | | | | *P-*value |
| --- | --- | --- | --- | --- | --- | --- |
|  | Total | 0-3 | 4 | 5 | ≥ 6 |  |
| Age (years) | 45.5 (0.3) | 43.4 (0.5) | 44.4 (0.5) | 44.9 (0.5) | 48.3 (0.5) | <0.001 |
| Sex,  n (weighted %) |  |  |  |  |  | 0.002 |
| Female | 4,021 (48.0) | 861 (44.3) | 991 (46.6) | 921 (48.6) | 1,248 (51.3) |  |
| Male | 4,379 (52.0) | 1,107 (55.7) | 1,071 (53.4) | 1,014 (51.4) | 1,187(48.7) |  |
| Race, n (weighted %) |  |  |  |  |  | <0.001 |
| Mexican American | 1,210 (7.7) | 281 (8.4) | 345 (9.5) | 296 (8.4) | 288 (5.3) |  |
| Non-Hispanic Black | 1,602 (10.2) | 497 (14.2) | 410(11.4) | 366 (10.3) | 329 (6.5) |  |
| Non-Hispanic White | 3,810 (70.0) | 830 (66.5) | 869 (65.8) | 850 (68.4) | 1,261 (76.8) |  |
| Other Race | 1,778 (12.1) | 360 (10.9) | 438 (13.3) | 423 (12.9) | 557 (11.4) |  |
| PIR | 3.10 (0.04) | 2.79 (0.06) | 2.87 (0.06) | 3.22 (0.06) | 3.42 (0.05) | <0.001 |
| BMI (kg/m^2)^ | 28.69 (0.12) | 29.58 (0.19) | 28.93 (0.20) | 28.72 (0.22) | 27.86 (0.18) | <0.001 |
| Educational level,  n (weighted %) |  |  |  |  |  | <0.001 |
| Less than high school | 579 (3.4) | 143 (3.8) | 176 (4.5) | 143 (3.6) | 117 (2.0) |  |
| High school or equivalent | 2,897 (31.2) | 843 (40.7) | 764 (34.3) | 640 (28.5) | 650 (23.9) |  |
| College or above | 4,924 (65.5) | 982 (55.5) | 1,122 (61.3) | 1,152( 67.8) | 1,668 (74.1) |  |
| Marital status,  n (weighted %) |  |  |  |  |  | 0.009 |
| Widowed/Divorced/Never married | 3,289 (35.5) | 809 (37.9) | 856 (37.9) | 750 (35.6) | 874 (31.8) |  |
| Married/Living with a partner | 5,111 (64.5) | 1,159 (62.1) | 1,206 (62.1) | 1,185 (64.4) | 1,561 (68.2) |  |
| Smoking status,  n (weighted %) |  |  |  |  |  | <0.001 |
| Never | 4,672 (56.0) | 1,047 (55.1) | 1,111 (53.9) | 1,099 (57.6) | 1,415 (56.9) |  |
| Former | 2,058 (25.3) | 456 (23.0) | 480 (24.1) | 458 (23.5) | 664 (29.1) |  |
| Now | 1,670 (18.8) | 465 (21.9) | 471 (22.0) | 378 (18.9) | 356 (14.0) |  |
| Alcohol drinking,  n (weighted %) |  |  |  |  |  | <0.001 |
| Former | 1,128 (10.8) | 289 (12.0) | 280 (11.0) | 260 (11.2) | 299 (9.5) |  |
| Mild | 3,083 (39.3) | 645 (34.8) | 689 (35.0) | 693 (37.9) | 1,056 (46.7) |  |
| Moderate | 1,378 (18.3) | 332 (18.3) | 325 (16.9) | 316 (18.8) | 405 (19.0) |  |
| Heavy | 1,839 (22.6) | 494 (25.8) | 495 (25.7) | 435 (23.5) | 415 (17.2) |  |
| Never | 972 (9.0) | 208 (9.1) | 273 (11.3) | 231 (8.5) | 260 (7.6) |  |
| Physical activity,  n (weighted %) |  |  |  |  |  | 0.010 |
| Mild | 1,548 (16.9) | 353 (17.6) | 373 (16.8) | 360 (17.0) | 462 (16.4) |  |
| Moderate | 3,376 (41.2) | 721 (37.7) | 793 (39.0) | 791 (41.5) | 1,071 (45.1) |  |
| Vigorous | 3,476 (41.9) | 894 (44.8) | 896 (44.2) | 784 (41.5) | 902 (38.5) |  |
| Hypertension,  n (weighted %) |  |  |  |  |  | 0.124 |
| No | 5,145 (65.5) | 1,182 (64.9) | 1,259 (63.6) | 1,227 (68.3) | 1,477 (65.3) |  |
| Yes | 3,255 (34.5) | 786 (35.1) | 803 (36.4) | 708 (31.7) | 958 (34.7) |  |
| Diabetes,  n (weighted %) |  |  |  |  |  | 0.077 |
| No | 6,898 (86.9) | 1,574 (85.2) | 1,667 (85.9) | 1,612 (88.2) | 2,045 (87.9) |  |
| Yes | 1,502 (13.1) | 394 (14.8) | 395 (14.1) | 323 (11.8) | 390 (12.1) |  |
| CVD, n (weighted %) |  |  |  |  |  | 0.460 |
| No | 7,650 (92.7) | 1,777 (92.1) | 1,878 (93.0) | 1,751 (92.2) | 2,244 (93.4) |  |
| Yes | 750 (7.3) | 191 (7.9) | 184 (7.0) | 184 (7.8) | 191 (6.6) |  |
| CKD, n (weighted %) |  |  |  |  |  | 0.535 |
| No | 7,204 (88.7) | 1,673 (88.5) | 1,753 (87.8) | 1,674 (88.7) | 2,104 (89.4) |  |
| Yes | 1196 (11.3) | 295 (11.5) | 309 (12.2) | 261 (11.3) | 331(10.6) |  |
| SBP (mmHg) | 120.16 (0.26) | 120.92 (0.51) | 120.59 (0.50) | 119.46 (0.49) | 119.81 (0.48) | 0.168 |
| DBP (mmHg) | 70.10 (0.24) | 69.96 (0.45) | 70.29 (0.32) | 70.08 (0.30) | 70.07 (0.34) | 0.790 |
| HbA1c (%) | 5.56 (0.01) | 5.60 (0.02) | 5.60 (0.02) | 5.54 (0.02) | 5.52 (0.02) | 0.011 |
| Cholesterol (mmol/L) | 4.98 (0.02) | 4.93 (0.03) | 4.96 (0.03) | 5.00 (0.04) | 5.01 (0.03) | 0.253 |
| SCR (umol/L) | 77.09 (0.34) | 78.70 (0.89) | 77.42 (0.70) | 76.71 (0.55) | 75.95 (0.48) | 0.024 |
| CKM, n (weighted %) |  |  |  |  |  | 0.001 |
| Non–CKM syndrome | 810 (11.7) | 161 (9.2) | 194 (10.8) | 186 (12.0) | 269 (14.0) |  |
| CKM syndrome | 7,590 (88.3) | 1,807 (90.8) | 1,868 (89.2) | 1,749 (88.0) | 2,166 (86.0) |  |

Note: Continuous variables are described as weighted means (standard errors). For categorical variables, unweighted N reflect the study sample, while percentages represent survey-weighted values.

*Abbreviations*: DI-GM, dietary index for gut microbiota; PIR, poverty income ratio; BMI, body mass index; CVD, cardiovascular disease; CKD, chronic kidney disease; SBP, systolic blood pressure, DBP, diastolic blood pressure; HbA1c, glycosylated hemoglobin; SCR, serum creatinine; CKM, cardiovascular‐kidney‐metabolic.

**Supplementary Table 3** Univariable weighted logistic regression analysis of CKM syndrome compared with non-CKM syndrome.

| Characteristics | OR (95% CI) | *P-*value |
| --- | --- | --- |
| Age | 1.06 (1.05, 1.07) | <0.001 |
| Sex |  |  |
| Female | Reference | Reference |
| Male | 2.19 (1.83, 2.62) | <0.001 |
| Race |  |  |
| Mexican American | Reference | Reference |
| Non-Hispanic Black | 0.99 (0.68, 1.45) | 0.969 |
| Non-Hispanic White | 0.57 (0.42, 0.77) | <0.001 |
| Other Race | 0.56 (0.39, 0.79) | 0.001 |
| Marital status |  |  |
| Widowed/Divorced/Never married | Reference | Reference |
| Married/Living with a partner | 1.44 (1.21, 1.71) | <0.001 |
| Education |  |  |
| Less than high school | Reference | Reference |
| High school or equivalent | 0.54 (0.34, 0.87) | 0.012 |
| College graduate or above | 0.31 (0.19, 0.51) | <0.001 |
| PIR | 0.92 (0.87, 0.98) | 0.008 |
| Smoking status |  |  |
| Never | Reference | Reference |
| Former | 2.71 (2.11, 3.48) | <0.001 |
| Now | 1.40 (1.12, 1.76) | 0.004 |
| Alcohol drinking |  |  |
| Never | Reference | Reference |
| Former | 2.05 (1.33, 3.18) | 0.002 |
| Mild | 0.97 (0.68, 1.38) | 0.871 |
| Moderate | 0.77 (0.52, 1.14) | 0.186 |
| Heavy | 0.96 (0.67, 1.38) | 0.839 |
| Physical activity |  |  |
| Mild | Reference | Reference |
| Moderate | 0.58 (0.44, 0.75) | <0.001 |
| Vigorous | 0.63 (0.48, 0.84) | 0.002 |
| DI-GM score | 0.89 (0.84, 0.93) | <0.001 |
| DI-GM group |  |  |
| 0-3 | Reference | Reference |
| 4 | 0.84 (0.64, 1.09) | 0.176 |
| 5 | 0.74 (0.58, 0.95) | 0.017 |
| ≥ 6 | 0.62 (0.49, 0.78) | <0.001 |

*Abbreviations*: DI-GM, dietary index for gut microbiota; CKM, cardiovascular‐kidney‐metabolic; OR, odds ratio; CI, confidence interval; PIR, poverty income ratio.

**Supplementary Table 4** Subgroup analysis for the association between DI-GM and CKM syndrome.

| Subgroup | DI-GM levels | | | | | | | | | | |
| --- | --- | --- | --- | --- | --- | --- | --- | --- | --- | --- | --- |
|  | 0-3 | 4 |  |  | 5 |  |  | ≥ 6 |  |  |  |
|  | OR  (95% CI) | OR  (95% CI) | *P* -value | adjusted *P*-value | OR  (95% CI) | *P* -value | adjusted *P*-value | OR  (95% CI) | *P* -value | adjusted *P*-value | *P* for interaction |
| Age |  |  |  |  |  |  |  |  |  |  | 0.217 |
| 20-39 | Reference | 0.78 (0.56,1.09) | 0.142 | 1.278 | 0.90 (0.64,1.26) | 0.525 | 4.725 | 0.62 (0.46,0.85) | 0.004 | 0.036 |  |
| 40-59 | Reference | 1.02 (0.54,1.92) | 0.956 | 8.604 | 0.60 (0.31,1.15) | 0.121 | 1.089 | 0.50 (0.27,0.90) | 0.022 | 0.198 |  |
| ≥ 60 | Reference | 0.80  (0.17,3.61) | 0.764 | 6.876 | 0.62  (0.19,1.99) | 0.418 | 3.762 | 0.62 (0.22,1.73) | 0.353 | 3.177 |  |
| Sex |  |  |  |  |  |  |  |  |  |  | 0.004 |
| Female | Reference | 0.68 (0.45,1.01) | 0.055 | 0.33 | 0.49 (0.35,0.69) | <0.001 | <0.001 | 0.48 (0.32,0.72) | <0.001 | <0.001 |  |
| Male | Reference | 1.01 (0.67,1.50) | 0.98 | 5.88 | 1.43 (0.93,2.17) | 0.099 | 0.594 | 0.67 (0.45,1.00) | 0.049 | 0.294 |  |
| Race |  |  |  |  |  |  |  |  |  |  | 0.078 |
| Mexican American | Reference | 0.50 (0.25,0.98) | 0.043 | 0.516 | 0.62 (0.28,1.38) | 0.24 | 2.88 | 0.89 (0.42,1.90) | 0.763 | 9.156 |  |
| Non-Hispanic Black | Reference | 0.96 (0.49,1.88) | 0.907 | 10.884 | 1.25 (0.60,2.61) | 0.551 | 6.612 | 0.90 (0.45,1.79) | 0.765 | 9.18 |  |
| Non-Hispanic White | Reference | 0.85 (0.59,1.22) | 0.370 | 4.44 | 0.72 (0.50,1.02) | 0.066 | 0.792 | 0.52 (0.37,0.73) | <0.001 | <0.001 |  |
| Other Race | Reference | 0.98 (0.57,1.67) | 0.930 | 11.16 | 0.89 (0.51,1.58) | 0.697 | 8.364 | 0.81 (0.48,1.37) | 0.434 | 5.208 |  |
| Marital status |  |  |  |  |  |  |  |  |  |  | 0.483 |
| Widowed/Divorced/Never married | Reference | 0.85 (0.54,1.32) | 0.457 | 2.742 | 0.92 (0.58,1.46) | 0.719 | 4.314 | 0.56 (0.35,0.89) | 0.015 | 0.09 |  |
| Married/Living with a partner | Reference | 0.83 (0.57,1.22) | 0.340 | 2.04 | 0.66 (0.45,0.96) | 0.029 | 0.174 | 0.58 (0.41,0.84) | 0.004 | 0.024 |  |
| Educational levels |  |  |  |  |  |  |  |  |  |  | 0.201 |
| Less than high school | Reference | 0.47 (0.12, 1.77) | 0.256 | 2.304 | 0.37 (0.11,1.27) | 0.111 | 0.999 | 1.63 (0.25,10.79) | 0.604 | 5.436 |  |
| High school or equivalent | Reference | 0.80 (0.46,1.41) | 0.437 | 3.933 | 1.05 (0.62,1.77) | 0.866 | 7.794 | 0.73 (0.40,1.33) | 0.303 | 2.727 |  |
| College graduate or above | Reference | 0.82 (0.57,1.18) | 0.288 | 2.592 | 0.68 (0.50,0.93) | 0.017 | 0.153 | 0.53 (0.38,0.74) | <0.001 | <0.001 |  |
| PIR |  |  |  |  |  |  |  |  |  |  | 0.180 |
| ≤ 1.3 | Reference | 0.76 (0.42,1.35) | 0.342 | 3.078 | 1.33 (0.77,2.29) | 0.297 | 2.673 | 0.78 (0.43,1.41) | 0.412 | 3.708 |  |
| > 1.3 to ＜ 3.5 | Reference | 1.10 (0.69,1.75) | 0.683 | 6.147 | 0.88 (0.58,1.32) | 0.528 | 4.752 | 0.61 (0.38,0.98) | 0.040 | 0.360 |  |
| ≥ 3.5 | Reference | 0.67 (0.41, 1.11) | 0.118 | 1.062 | 0.54 (0.34,0.85) | 0.009 | 0.081 | 0.49 (0.29,0.81) | 0.006 | 0.054 |  |
| Physical activity |  |  |  |  |  |  |  |  |  |  | 0.113 |
| Mild | Reference | 0.61 (0.23, 1.64) | 0.321 | 2.889 | 0.35 (0.15,0.80) | 0.014 | 0.126 | 0.57 (0.21,1.54) | 0.261 | 2.349 |  |
| Moderate | Reference | 0.77 (0.47,1.28) | 0.311 | 2.799 | 0.60 (0.39,0.93) | 0.023 | 0.207 | 0.52 (0.32,0.83) | 0.007 | 0.063 |  |
| Vigorous | Reference | 0.88 (0.56,1.37) | 0.569 | 5.121 | 1.13 (0.69,1.87) | 0.615 | 5.535 | 0.60 (0.40,0.91) | 0.017 | 0.153 |  |

*Note*: Each stratification was adjusted for age, sex, race, marital status, educational levels, poverty income ratio, smoking behavior, alcohol consumption, physical activity, and total energy intake, unless the variable was already used as a stratification factor. *Abbreviations*: DI-GM, dietary index for gut microbiota; CKM, cardiovascular‐kidney‐metabolic; OR, odds ratio; CI, confidence interval; PIR, poverty income ratio.

**Supplementary Table 5** Weighted multivariable logistic regression analyses of DI-GM and CKM syndrome after interpolation of covariates.

| Characteristics | Model 1 | | Model 2 | | Model 3 | |
| --- | --- | --- | --- | --- | --- | --- |
|  | OR (95% CI) | *P* value | OR (95% CI) | *P* value | OR (95% CI) | *P* value |
| DI-GM score | 0.90 (0.86, 0.94) | <0.001 | 0.85 (0.81, 0.89) | <0.001 | 0.87(0.83,0.92) | <0.001 |
| DI-GM group |  |  |  |  |  |  |
| 0-3 | Reference | Reference | Reference | Reference | Reference | Reference |
| 4 | 0.83 (0.67, 1.04) | 0.110 | 0.81 (0.63, 1.03) | 0.081 | 0.84 (0.66, 1.07) | 0.157 |
| 5 | 0.74 (0.61, 0.91) | 0.005 | 0.70 (0.56, 0.88) | 0.002 | 0.75 (0.60, 0.94) | 0.015 |
| ≥ 6 | 0.65 (0.53, 0.79) | <0.001 | 0.53 (0.42, 0.66) | <0.001 | 0.60 (0.48, 0.75) | <0.001 |
| Trend test |  | <0.001 |  | <0.001 |  | <0.001 |

Note: Model 1 was not adjusted for any covariates.

Model 2 was adjusted for age, sex, and race/ethnicity.

Model 3 was adjusted for age, sex, race/ethnicity, marital status, education level, PIR, alcohol intake, smoking status, physical activity, and total energy intake.

*Abbreviations*: DI-GM, dietary index for gut microbiota; CKM, cardiovascular‐kidney‐metabolic; OR, odds ratio; CI, confidence interval; PIR, poverty income ratio.

**
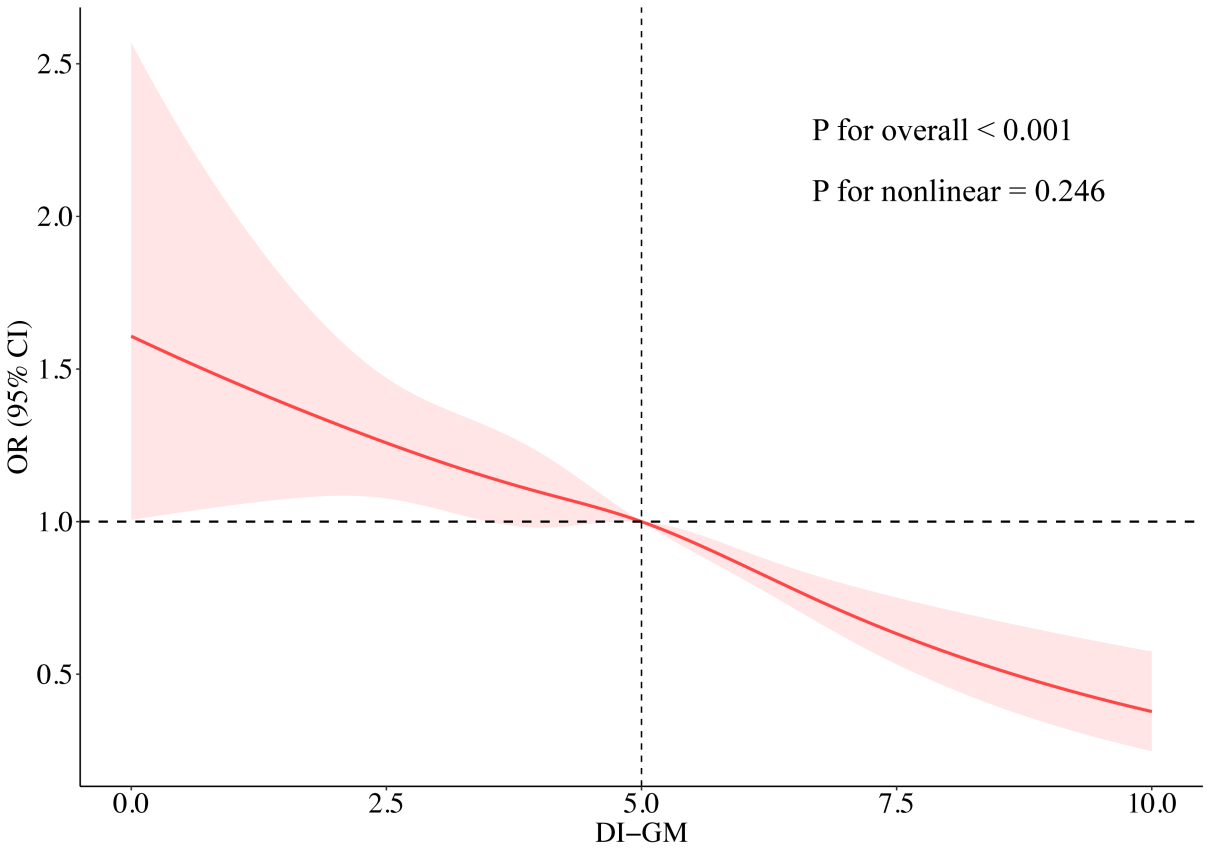
**

**Supplementary Figure 1** Association between DI-GM and the risks of CKM syndrome analyzed through a restricted cubic spline model.

Note: The restricted cubic spline model was adjusted for age, sex, race/ethnicity, marital status, education level, PIR, alcohol intake, smoking status, physical activity, and total energy intake.

*Abbreviations*: DI-GM, dietary index for gut microbiota; CKM, cardiovascular‐kidney‐metabolic; OR, odds ratio; CI, confidence interval.
